# Evaluation of the Submicron Particles Distribution Between Mountain and Urban Site: Contribution of the Transportation for Defining Environmental and Human Health Issues

**DOI:** 10.3390/ijerph16081339

**Published:** 2019-04-14

**Authors:** Maurizio Manigrasso, Carmela Protano, Stefano Martellucci, Vincenzo Mattei, Matteo Vitali, Pasquale Avino

**Affiliations:** 1 Department of Technological Innovations, National Institute for Insurance against Accidents at Work INAIL, via IV Novembre 144, I-00187 Rome, Italy; m.manigrasso@inail.it; 2 Department of Public Health and Infectious Diseases, University of Rome “La Sapienza”, p.le Aldo Moro 5, I-00185 Rome, Italy; carmela.protano@uniroma1.it (C.P.); matteo.vitali@uniroma1.it (M.V.); 3 Laboratory of Experimental Medicine and Environmental Pathology, Polo Universitario di Rieti, Sabina Universitas, I-02100 Rieti, Italy; s.martellucci@sabinauniversitas.it (S.M.); vincenzo.mattei@uniroma1.it (V.M.); 4 Department of Agricultural, Environmental and Food Sciences (DiAAA), University of Molise, via De Sanctis, I-86100 Campobasso, Italy; 5 Institute of Ecotoxicology & Environmental Sciences, Kolkata 700156, India

**Keywords:** submicron particles, mountain and urban site, transportation, human health

## Abstract

Transportation is one of the main causes of atmospheric pollution, especially in downtown big cities. Researchers usually point their attention to gaseous and/or particulate matter pollutants. This paper investigated the role of submicron particles, particularly the fraction ranging between 5–560 nm, in aerosol chemistry for identifying the contribution of autovehicular traffic and investigating the doses deposited in the human respiratory tract. Measurements carried out by two Fast Mobility Particle Sizer (FMPS, TSI) analyzers were simultaneously performed at two different sampling sites (an urban and a mountain site) during workdays and weekends in July. The total particle number (2–2.5 times higher in the urban site), the aerosol size distribution (different modes during the day), and the ultrafine/non-ultrafine particle ratios (ranging between 2–4 times between two sites) were investigated and discussed in relationship to the high autovehicular traffic in Rome and the almost null anthropogenic emissions at the mountain site, as well as the differing contributions of both to the “fresh nucleation” and to “aged aerosol”. Furthermore, the regional cumulative number doses deposited in the human respiratory tract were studied for both sites: The difference between the urban/mountain site was very high (up to 15 fold), confirming the pollutant role of transportation.

## 1. Introduction

Although urban areas occupy only 2% of the planet’s surface, they are primarily responsible for atmospheric pollutant emissions (e.g., about 80% of CO_2_ worldwide) as over half of the human population lives in such areas [1]. This occurrence simultaneously means a strong driving force for economic development and an excessive exploitation of resources and the environment, degradation and congestion. In Italy, the demographic trend is similar to the global one; urban areas represent 3.3% of the country whereas 23.4% of the population reside in such areas and this concentration affects various factors, particularly those of public health [2]. In 2005, the Position Paper [3] on particulate matter (PM) recommended the employment of PM_2.5_ for evaluating population exposure and in 2015 the International Agency for Research on Cancer (IARC) appointed PM [4,5,6,7] as a leading environmental cause of cancer deaths and classified it as carcinogenic to humans in Group I. Over the last decade, different European projects (for instance, EXPOLIS, RUPIOH, TRAPCA, AIRALLERG, HEPMEAP, PAMCHAR, HEAPPS, just to cite a few) focused both on classical Particulate Matter (PM) fractions (i.e., PM_10_ and PM_2.5_) and on new classifications (PM_1_ and, mainly, the submicron fraction), studying the number concentration and size distribution in urban areas. It should be considered that PM_2.5_ and PM_10_ are not two independent exposure indicators, because the second “contains” the first by definition [8]. Moreover, PM_2.5_ averagely constitutes about 70% of PM_10_ or PM_2.5_/PM_10_ is about 0.7 [9]; the daily observations of the two fractions underline a noteworthy inter-day and seasonal variability of this relationship, and suggests possible geographical differences. On the other hand, both in urban areas and in rural areas, the particle number concentrations show values ranging from a few hundred, up to 10^7^–10^8^ particles cm^−3^. Considering this wide dimensional range, it is important to understand under what concentration levels occur and which aerosol size distributions are.

The literature reports numerous studies carried out worldwide, showing how the numerical concentration and the particle size distribution vary according to multiple factors. Minoura and Takekawa carried out an aerosol monitoring in an urban area in Japan [10]. They obtained average particle values in the range 13.6–685.4 nm, in two different seasons. In the summer season, the values were about 2800 particles cm^−3^, whereas in winter the value was about six times as much (18,000 particles cm^−3^). During the winter the peak, concentration was recorded around 40 nm in the morning rush hours, whereas in other hours of the day the average diameter moved to 70 nm. Stanier et al. carried out a study in an urban area and in a rural area of Pittsburgh for one year [11]. Overall, in the urban area the average particle number in the size range 3–560 nm was 22,000 particles cm^−3^ (with a peak around 40 nm), whereas in rural areas the average value was 2–3 times lower. Hussein et al. carried out measurements of the particle size distribution in the urban (8–400 nm) and suburban (3–400 nm) areas of Helsinki [12]. The particle number was higher in urban locations with concentration peaks reaching values of 140,000 particles cm^−3^, whereas in suburban areas the values varied from 3000 up to 32,000 particles cm^−3^. The ultrafine particles (<100 nm, UFPs) contributed for more than 90% of the total concentration in urban areas, and for 70%–80% in suburban areas. Furthermore, near busy roads the average particle number concentration could exceed 60,000 particles cm^−3^ (60% is represented by particles of the nucleation interval). The highest concentration values were recorded during peak hours (07:00–11:00), whereas the lower ones were recorded during the early hours of the day (02:00–05:00).

Different studies have shown that the PM_2.5_ fraction is associated with the increase of pathologies and deaths due to cardiovascular and/or respiratory disorders. With regard to submicron particles and UFPs, some effects on the human health have been highlighted, but these fractions represent a research line that still needs further epidemiological and toxicological studies. Particularly, the aerosol fraction below 50 nm shows high mobility in the atmosphere and can be inhaled easily by humans through the respiratory tract. While PM_2.5_ is deposited along the respiratory tract, a very large number of nanometer-sized particles can reach the pulmonary alveoli. Furthermore, some studies have shown a higher mutagenicity and an increased risk for humans exposed to nanoparticles. For these reasons, the monitoring of the submicron fraction and UFPs in the atmosphere should be considered in the regulation of air quality, as also suggested by recent studies [13].

This paper investigates the differences between submicron particle fractions investigated in downtown Rome, a big urban area characterized by transportation, and in a remote area, Monte Terminillo (Rieti, Italy) where no anthropogenic sources are present. For this reason, this remote site could be considered as the UFP “background”. The comparison between the two sites will be performed, taking into account the particle number concentrations and the size distributions. The differences will refer to the different contributions present in the relative areas. Furthermore, an evaluation of the doses deposited in the human respiratory tract based on the measurements performed in the two sites will be estimated for evaluating the impact of anthropogenic sources on the human health.

## 2. Materials and Methods

The methodology used is based on performing simultaneous determinations in two different sites and relies on the aerosol aerodynamic-diameter analysis for evidencing the contribution of traffic emission to the submicron particle fraction.

### 2.1. Sampling Sites

**Figure 1:**
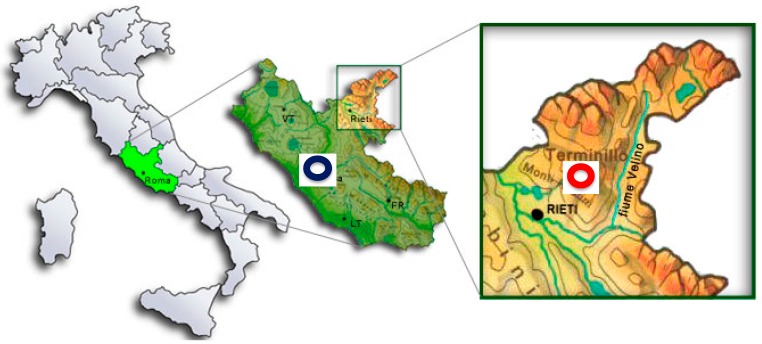
Rome (blue circle) and Monte Terminillo (red circle) in the Latium region.

Submicron particle measurements were carried out in downtown Rome and at Monte Terminillo. Figure 1 shows the locations of Rome and Monte Terminillo in the Latium region (Central Italy): Rome is 30 km from the sea, whereas Monte Terminillo is on the eastern part of the region.

In Rome, the sampling site was located at INAIL’s Pilot Station (41°53′46″ N, 12°29′46″ E), the sampling point was at 3 m height from the ground. The site is characterized by a high density (ratio of 706 vehicles per 1000 inhabitants, source Automobile Club d’Italia, ACI) of autovehicular traffic (e.g., cars, motorbikes, buses, pullmans), whereas the street could be assessed like a canyon (height/width ratio 3:1 between buildings and street). Furthermore, according to recent data on vehicle fuels (source ACI), almost 40% of the total park was diesel.

Monte Terminillo (42°28′23.77″ N, 12°59′50.35″ E), a massif in the Monti Reatini, Central Italy, is located some 20 km from Rieti and 100 km from Rome with a high altitude of 2217 meters (7274 ft), and the temperature ranged between 10 °C and 17 °C with a relative humidity of 70% during the entire investigated period. No rainy day was recorded during the campaign. There were two different ways to reach Monte Terminillo by car but access was limited and the anthropogenic emissions were basically absent. Monte Terminillo could be considered a remote site.

### 2.2. Measurement Equipment

Particle number size distributions at high time-resolution (1 s time) were carried out by means of a Fast Mobility Particle Sizer (FMPS, model 3091, TSI, Shoreview, MN, USA) with a range from 5.6 to 560 nm electrical mobility diameter. The instrument worked at 10 L·min^−1^ for minimizing the diffusion UFP losses and counts and classifies the particles in 32 size channels. The FMPS performance was investigated by comparing it with a Scanning Mobility Particle Sizer (SMPS) [14,15]. The measurement campaign was simultaneously performed in both sites (Rome and Monte Terminillo) and was three weeks long and carried out in July 2018.

### 2.3. Dose Deposition Model

Particle deposition in the human respiratory system was evaluated using the Multiple-Path Particle Dosimetry model (MPPDv3.01, ARA 2015, ARA, Arlington, VA, USA) [16]. The 60th percentile human stochastic lung was considered along with the following settings: (i) A uniformly expanding flow, (ii) upright body orientation, and (iii) nasal breathing with a 0.5 inspiratory fraction and no pause fraction. Moreover, the following parameters were used for a Caucasian adult male under light work physical activity, based on the ICRP report [17,18,19,20]: (i) A functional residual capacity (FRC) of 3300 mL, (ii) an upper respiratory tract (URT) volume equal to 50 mL, (iii) a 20 min^−1^ breathing frequency, and (iv) an air volume inhaled during a single breath (tidal volume, V_t_) of 1.25 L.

Since FMPS measures aerosol size number distribution as a function of the electrical mobility diameter (*d*), *d* values were transformed to aerodynamic diameter (*d_a_*) according to Equation (1) [21]:

(1) $$d_{a}=d\sqrt{\chi \times \frac{\rho \times C_{c}\left(d_{m}\right)}{C_{c}\left(d_{a}\right)}}$$

where *C_c_* is the Cunningham slip factor for a given diameter, ρ is the particle density, and χ is the particle dynamic shape factor. A total of 1.5 g cm^−3^ particle density was assumed. χ as a function of *d_a_* was estimated by interpolating, through a cubic spline function [22] with the data reported by Hu et al. [23] in the range from 0.1 to 1.8 µm in Beijing. For *d_a_* below this range, the relevant lower bound χ values were adopted.

For each respiratory act, the doses described below were calculated as regional dose size number distributions (*D^R^*) as function of time (t) in the head (H), tracheobronchial (TB), and alveolar (Al) regions (R):

(2) $$D^{R}\left(d_{i},t\right)=F^{R}\left(d_{i}\right)\times C\left(d_{i},t\right)\times V_{t}\;\;\;(R\;=\;H,\;\mathrm{TB},\;\mathrm{Al})$$

where *d_i_* is the diameter of particles classified in the i^th^ FMPS size channel, *F^R^(d_i_)* is the relevant deposition fraction at a given *R* region, *C(d_i_,t)* is the concentration of particles in the i^th^ FMPS size channel as a function of time, and *V_t_* is the tidal volume.

Average regional and total size number dose distributions over 1h time interval Δ*t*:

(3) $$\bar{D^{R}}\left(d_{i},\Delta t\right)=F^{R}\left(d_{i}\right)\times \bar{C}\left(d_{i},\Delta t\right)\times V_{t}$$

(4) $$\bar{D^{Tot}}\left(d_{i},\Delta t\right)=\underset{R}{\sum }\bar{D^{R}}\left(d_{i},\Delta t\right)$$

where $\bar{C}\left(d_{i},\Delta t\right)$ is the average concentration over the time interval Δ*t* of the particles in the i^th^ FMPS size channel.

Total regional number doses as functions of time:

(5) $$D^{R}\left(t\right)=\overset{32}{\underset{i=1}{\sum }}D^{R}\left(d_{i,}t\right)\;\;\;\left(R\;=\;H,\;\mathrm{TB},\;\mathrm{Al}\right)$$

where 32 is the number of FMPS size classes.

The total number dose in the respiratory system as functions of time:

(6) $$D^{Tot}\left(t\right)=\underset{R}{\sum }D^{R}\left(t\right)\;\;\;\left(R\;=\;H,\;\mathrm{TB},\;\mathrm{Al}\right)$$

Cumulative regional number doses and cumulative total number dose in the respiratory system were calculated over a 1 h time interval Δ*t*, according to Equations (5) and (6), respectively:

(7) $$D_{c}^{R}\left(t_{s}\right)=\overset{t_{s}}{\underset{t=t_{0}}{\sum }}D^{R}\left(t\right)\;\;\;\left(R\;=\;H,\;\mathrm{TB},\;\mathrm{Al}\right)$$

(8) $$D_{c}^{Tot}\left(t_{s}\right)=\overset{t_{s}}{\underset{t=t_{0}}{\sum }}D^{Tot}\left(t\right)$$

## 3. Results

The number concentrations and the relative aerosol size distributions can show different values in different environmental conditions. The meteorology, the daytime period, and the possible presence of local emission sources close to the monitoring site are parameters significantly affecting aerosol size distribution. In this study, according to the description reported in the previous section, the sampling site in Rome allowed to minimize the influence of weather conditions. Furthermore, the measurements performed only in the summer period allowed the study to not consider the domestic heating sources, which are an extremely important contribution to the aerosol. Therefore, the only contribution to aerosol came from autovehicular traffic. This source was quite null in the Monte Terminillo site whereas it was strong in the Rome site.

### 3.1. Particle Number Concentration

Preliminary data evaluation is shown in Table 1 where the number particle concentration data (5.6–560 nm) obtained in the two sites are shown (to be noted that for workdays the authors mean the week period from Monday to Friday, whereas for weekends the authors mean Saturday and Sunday).

**Figure 2:**
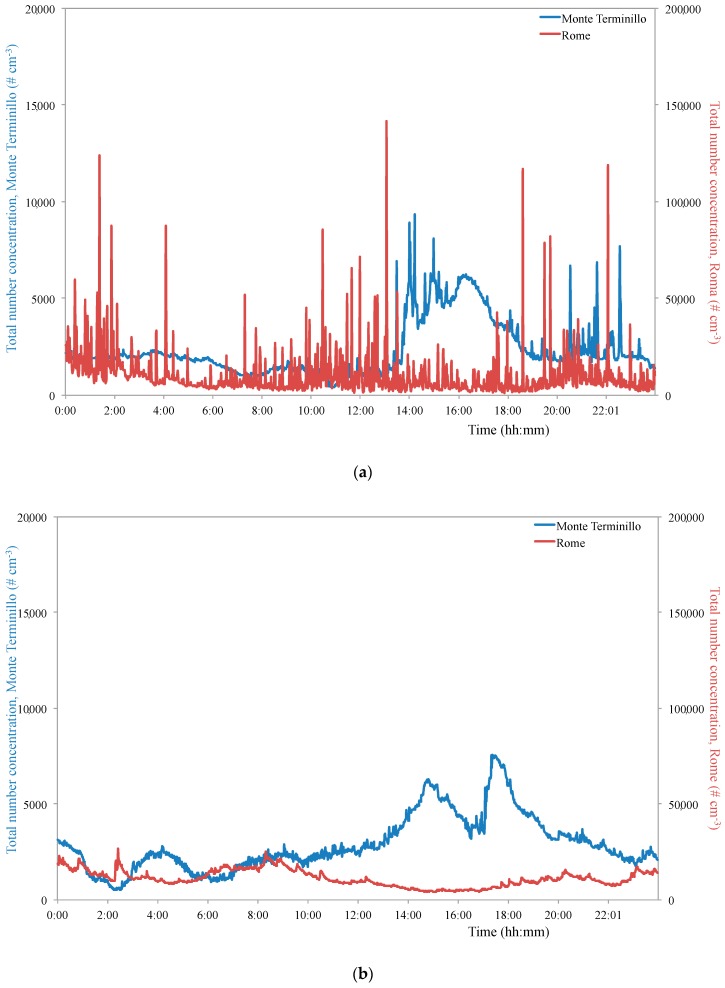
Typical number concentration daily trends recorded on workdays (**a**) and weekends (**b**) in the two sampling sites during the campaign.

Table 1 shows the total number concentration data along with a subdivision in ultrafine particles (UFPs, 5.6–99.3 nm) and in non-ultrafine particles (non-UFPs, 99.3–560 nm). Looking at the data, it can be seen that the total particles in the urban site averaged twice the level recorded at the mountain site during workdays and about 2.5 times on weekends. Similar ratios are observed for UFPs on workdays and weekends. On the other hand, non-UFPs show different relationships; during workdays the average ratio between the urban site and the mountain site was about 4, whereas during weekends it fell to 1.2. Thus, over the primary origin of such particles, it should also consider the physical-chemical processes leading to the novel particle formation starting from precursors in vapor or gas phase and from transformation processes: These phenomena are more incisive with high intensity of emission sources, i.e., autovehicular traffic. The measurements 95th percentile value confirms this occurrence. Although the average values on workdays and weekends determined in the Rome site were quite similar, as well as the maximum total particle value being about 7 times higher on weekends than on workdays, 95% of workday measurements were less than 26,930 # cm^−3^ whereas only 5% of the weekend measures exceeded 18,615 # cm^−3^ (Figure 2).

### 3.2. Human Respiratory Doses

**Figure 3:**
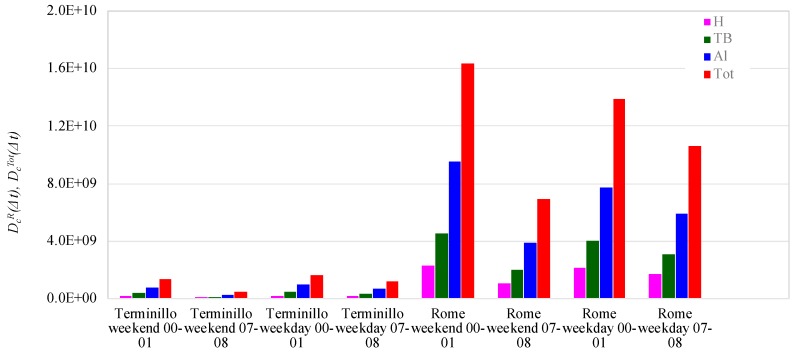
Cumulative regional number doses (*D_C_^R^(Δt)*) and cumulative total number dose (*D_C_^Tot^(Δt)*) in downtown Rome and in the Terminillo area, during workdays and weekends in the 00:00–01:00 and 07:00–08:00 1h-time intervals (head H, tracheobronchial TB, alveolar Al regions and total Tot).

Figure 3 describes the cumulative number doses (*D_c_^R^(Δt)*) deposited in the 00:00–01:00 and 07:00–08:00 time intervals, in the H, TB, and Al regions, as well as the relevant total doses (*D_c_^Tot^(Δt)*), estimated for the Rome and Terminillo measuring sites during workdays and holidays.

The two Δ*t* time intervals were chosen in order to evidence the effects of the emission source intensities and of the planetary boundary layer (mixing height) on aerosol concentrations and hence on the relevant respiratory doses. In the 07:00–08:00 time interval, the contribution of vehicular traffic in Rome started increasing, whereas in the Terminillo area aerosol emissions, apart from biogenic ones, were almost negligible. From 00:00 to 01:00, Rome traffic intensity decreased and in the Terminillo area, the contribution of biomass burning ceased being active. In this time interval, the Planetary Boundary Layer (PBL) mixing height was shallower and the pollutants tended to concentrate.

At the Terminillo and Rome sites, *D_c_^R^(Δt)* (R = H, TB, Al regions) and *D_c_^Tot^(Δt)*, varied respectively in the range 7.8 × 10^7^–2.2 × 10^8^, 1.3 × 10^8^–4.4 × 10^8^, 2.4 × 10^8^–9.6 × 10^8^, 4.5 × 10^8^–1.6 × 10^9^ particles and 1.0 × 10^9^–2.3 × 10^9^, 2.0 × 10^9^–4.5 × 10^9^, 3.8 × 10^9^–9.5 × 10^9^, 6.9 × 10^9^–1.6 × 10^10^ particles.

**Figure 4:**
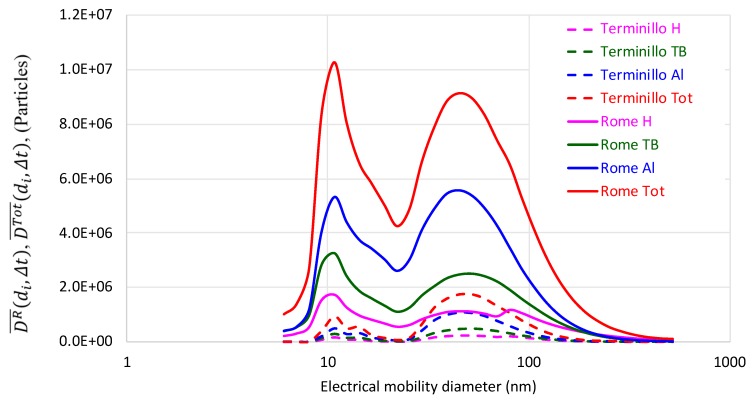
Average regional ($\bar{D^{R}}\left(d_{i},\Delta t\right)$) and total $\bar{D^{Tot}}\left(d_{i},\Delta t\right)$) size number dose distributions in the 07:00–08:00 1h-time interval in downtown Rome and in the Terminillo area.

Figure 4 shows the average regional ($\bar{D^{R}}\left(d_{i},\Delta t\right)$) and total $\bar{D^{Tot}}\left(d_{i},\Delta t\right)$) size number dose distributions in the 07:00–08:00 time interval. In both areas, bimodal size distributions, with modes at about 10 and 50 nm, were estimated.

## 4. Discussion

### 4.1. Particle Size Distribution

A complete and scrupulous aerosol size distribution should be based on the attribution of a (channel) size to each particle but the result should be extremely uncomfortable and not very usable. Therefore, the entire particle range (<1000 nm) was divided into a limited size range for measuring the particle number concentration in each size channel [24,25,26,27,28], i.e., the aerosol particle diameters were distributed in a rather wide range and their number concentrations vary considerably depending on the size. It should be noted that, in the aerosol size distribution studies performed in urban and suburban sites in the presence of traffic emissions, the particle classifications were observed even in more than three intervals [29]. For a better size distribution discussion, the authors considered the atmospheric submicron aerosol divided into three size ranges defined as follows:

- Nucleation range (~3–25 nm);
- Aitken nuclei range (~25–90 nm);
- Accumulation mode (~90–1000 nm; this paper studied particles in the range ~90–560 nm).

The nucleation range was defined below 25 nm, with the upper limit ranging between 20 and 30 nm [30,31,32]. The particles in the nucleation mode can derive both from direct emission and from formation processes, i.e., conversion reactions due to:

- Rapid cooling and dilution of gases and/or vapors produced by emissions;
- Chemical reactions involving precursors already present in the atmosphere.

**Figure 5:**
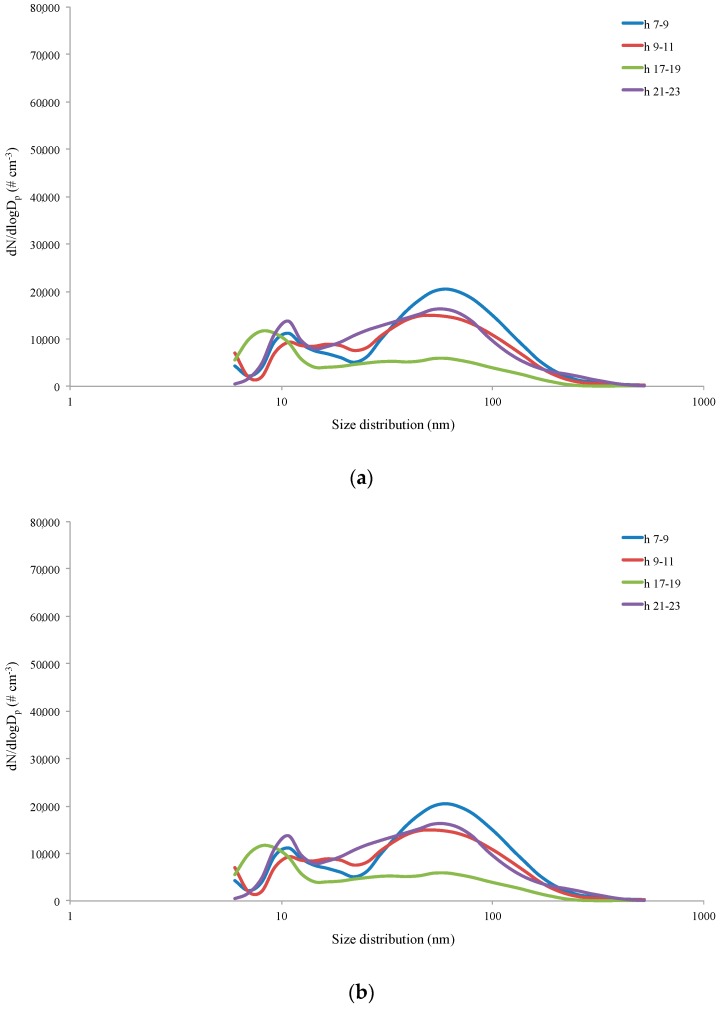
Typical daily size distribution in downtown Rome on weekdays (**a**) and weekends (**b**).

In agreement to what has been reported in the literature [33], high particle concentrations in nucleation mode were shown in the early hours of the morning, i.e., in the presence of high emission sources (autovehicular traffic). These particles tended to rapidly decrease spatially and temporally. The average daily particle size distribution in the nucleation mode showed a peak centered between 7 and 16 nm during workdays and between 8 and 14 nm during weekends in downtown Rome (Figure 5). Once formed and suspended in the atmosphere, the particles in the nucleation mode were characterized by Brownian motions: The collisions generated aggregation processes, increasing the particles in the nucleation mode up to the accumulation mode. It should be noted that the levels were different depending on the different intensity of the emission sources during the day.

On the other hand, aerosol in the range 25–90 nm (i.e., the Aitken nuclei range) comes from coagulation and condensation processes of particles in nucleation mode, but it can also be (produced and) directly emitted into the atmosphere from combustion sources. Particles in the accumulation mode are generally made up of carbon compounds, such as soot or dust. These can derive both from fuel engine combustion and from lubricating diesel or petrol oils, as well as from coagulation processes of particles in nucleation mode [34]. The “accumulation” aerosol (i.e., aerosol in accumulation mode) has a long lifetime in the atmosphere and can be transported over long distances as well.

**Figure 6:**
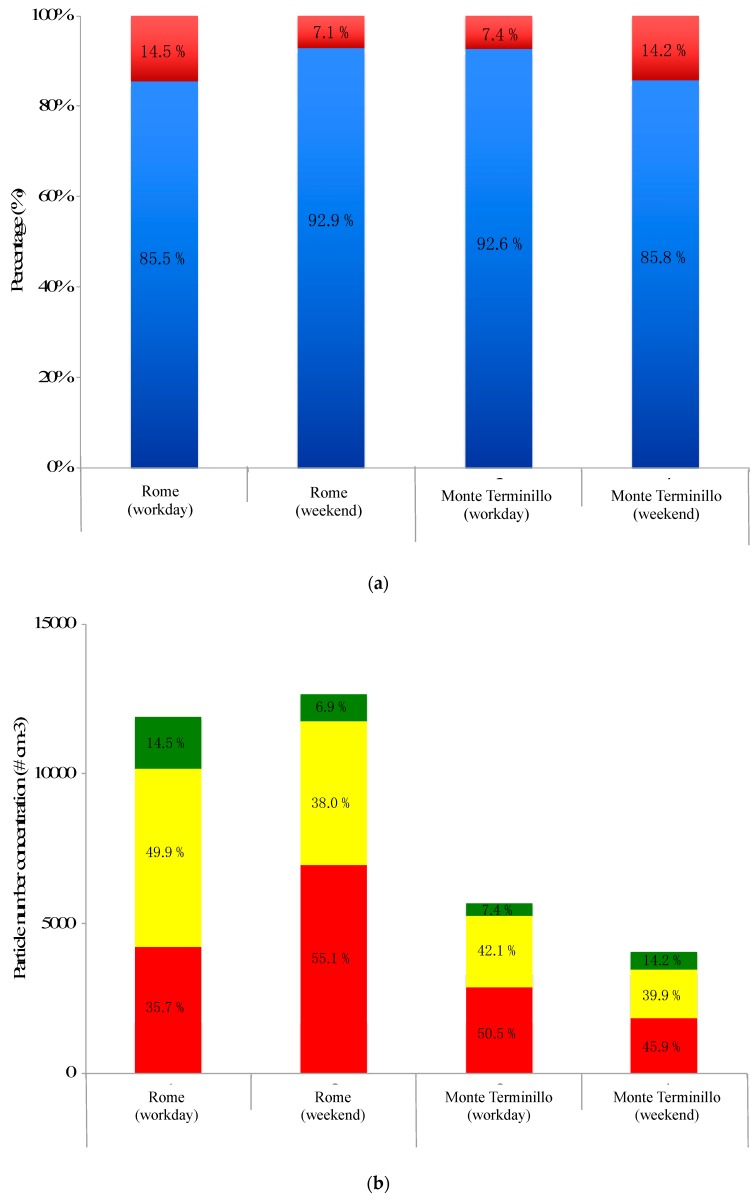
(**a**) UFPs and non-UFPs percentage ratio in the two sites during workdays and weekends; (**b**) particle number concentrations and average percentages of the submicron aerosol subdivided in three modes, i.e., nucleation mode (red bars), Aitken nuclei mode (yellow bars), and accumulation mode (green bars), in the two sites investigated.

Figure 6a shows the average UFP percentage to be predominant with respect to the relative percentage of non-UFPs in both sites both on workdays and weekends. A more detailed subdivision in the aforementioned size ranges makes it possible to highlight further considerations (Figure 6b).

Particles in nucleation mode are predominant in the mountain site, whereas in downtown Rome their percentage depends on the emission source intensity: They represent ~35% on workdays whereas they are ~55% on weekends. On the other hand, the accumulation mode becomes relevant during workdays in the urban area. In fact, it was about twice recorded during weekends. The particles were continuously emitted into the atmosphere by autovehicular traffic but at the same time they aggregated and/or coagulated and increased both the Aitken mode (small contribution) and the accumulation mode. In the Monte Terminillo site, the average particle number percentages both in nucleation mode and in the Aitken nuclei remained quite constant, whereas there was a noticeable increase in accumulation mode. This increase was opposite to what happened in the urban site but leads to the same considerations. The explanation is that during weekends the Monte Terminillo site could be reached by tourist coaches: access was limited but emissions from bus traffic strongly affected the ratio. The daily particle number concentration in the three subdivisions shows more than 2–3 times the value in the urban site than in the mountain site (Figure 6b).

**Figure 7:**
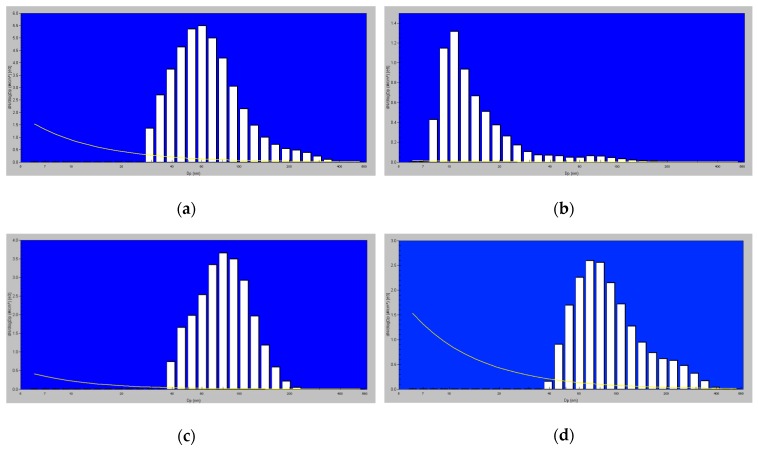

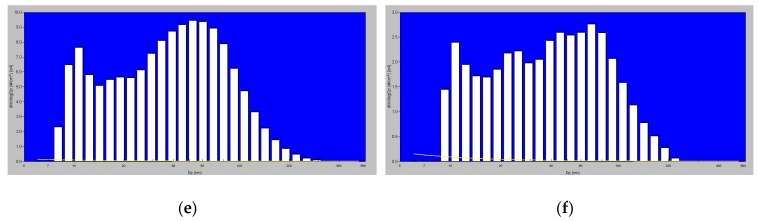
Typical aerosol distribution profiles determined (**a**,**b**) in the morning (07:00–09:00), (**c**,**d**) in rush hours (17:00–19:00), and (**e**,**f**) in the evening (20:00–22:00) during workdays at the Monte Terminillo site and in downtown Rome, respectively.

The difference between the two sites is evident in the aerosol size distributions reported in Figure 7a,b. Figure 7a,b show typical size distributions in the workday morning (07:00–09:00) at the Monte Terminillo site and in downtown Rome, respectively. In addition to the differences in the particle number (full-scale 6 × 10^3^ for the Monte Terminillo site, full-scale 1.4 × 10^5^ for downtown Rome), the size distribution profiles were different: In the urban area the mode between 7 and 14 nm was prevalent, meaning fresh particles emitted by vehicular traffic (“fresh nucleation”). This occurrence confirmed what has been reported in the literature; in the atmosphere most of the nanometer-sized particles (d ≤ 20 nm) mainly derive from gas-particle conversion processes [35], among which homogeneous (nucleation in vapor phase) and heterogeneous (nucleation in different phases) nucleation phenomena. The typical distribution recorded at the Monte Terminillo site showed a mono-mode centered around 60 nm. Figure 7c,e show the typical particle size profiles recorded at the Monte Terminillo site during rush hours and in the evening on workdays: It can be noted how both profiles are characterized by a single mode centered around 80–100 nm (with the full scale at 3 × 10^3^ particles cm^−3^). A different situation is evidenced in the Rome sampling site. Figure 7e shows the typical profile of the aerosol size distribution recorded during rush hours on workdays (full scale up to 10 × 10^4^ particles cm^−3^), i.e., during maximum intensity of autovehicular traffic (between 17:00–19:00). It can be noted that two modes, the first centered around 10 nm and the second between 30 and 110 nm. This second mode is an index of aged aerosol (“aged nucleation”) [29]. A similar consideration can be drawn for Figure 7f (full scale up to 3 × 10^4^ particles cm^−3^), where a typical size distribution occurring on the weekend evening in Rome is reported.

### 4.2. Dose Evaluation

Cumulative regional number doses (*D_C_^R^(Δt)*) and cumulative total number doses (*D_C_^Tot^(Δt)*) were from about 8 to 15 fold higher in downtown Rome than in the Terminillo area (Figure 3). At both sites *D_C_^R^(Δt)* and *D_C_^Tot^(Δt)*, doses were higher in the 00:00–01:00 interval than in the 07:00–08:00 1h time interval, suggesting that the lower nocturnal atmospheric mixing layer outweighed the traffic emission reduction in Rome. In the Terminillo area, the shallow nocturnal PBL mixing height was synergic with the biomass burning aerosol emissions that were active until 00:00. At both sites, the highest contribution derived from particles deposited in the Al region, followed by the TB and H doses, on average respectively about 56%, 29%, and 15% of the total doses deposited into the respiratory system at both sites.

Figure 4 shows that at both sites $\bar{D^{R}}\left(d_{i},\Delta t\right)$ and $\bar{D^{Tot}}\left(d_{i},\Delta t\right)$ were almost in the UFP size region (<100 nm). The average contributions of nucleation, Aitken, and accumulation mode particles to *D_C_^Tot^(Δt)* in downtown Rome and in the Terminillo area were respectively 55%, 37%, 8%, and 11%, 69%, and 20%. Nucleation mode particles brought about a higher contribution to total particle doses in Rome, due to the remarkable influence of freshly emitted traffic aerosol, typical of an urban area [36]. Finally, taking into account the literature, some considerations about the chemical composition and emissions sources in these areas could be drawn by measuring particle number and size. In urban areas, nucleation mode particles are mainly made of hydrocarbons, sulphates, and water and derives from vehicular exhaust dilution and cooling, whereas Aitken mode particles are mainly diesel soot particles [37,38] and accumulation mode particles include urban background and aged aerosol, road dust resuspension, and brake wear [39]. In the Terminillo area, nucleation and Aitken particles may derive from biogenic Volatile Organic Compounds (VOCs) emissions as well as from biomass burning, when this emission source is active. Such particles may grow into larger accumulation mode particles [40].

## 5. Conclusions

This study reported a deep comparison of the submicron aerosol fractions collected in a downtown big urban area (Rome), characterized by anthropogenic emissions, and a site (Monte Terminillo), where no anthropogenic emissions were recorded. Relevant differences both in the number particle concentrations and in the size aerosol distributions were found. Such divergences were essentially due to the different anthropogenic emissions between the two sites, particularly with autovehicular traffic. The study evidenced the different contribution of transportation to the local atmospheric pollution. This was quite evident in analyzing the aerosol profiles during nocturnal and diurnal profiles: The first ones were quite similar, whereas the second were different depending on the emission sources present in the two sites. These differences greatly reflected on human health and particularly on the cumulative regional/total number doses. A future study (actually, in preparation) will regard the synergic effect of transportation and domestic heating on aerosol composition, especially submicron fraction and UFPs, both in this remote location and in downtown Rome with the possibility to discern the two different contributions in aerosol fractions.

## Figures and Tables

**Table 1:** Typical average number concentration (# cm^−3^), standard deviation, and 95th percentile of total particles, ultrafine particles (UFPs), and non-ultrafine particles (non-UFPs) determined in the two sites during workdays and weekends.

Particles		Rome		Monte Terminillo	
		Workday	Weekend	Workday	Weekend
Total	average	11,905	10,006	5695	4072
	min–max	3058–35,964	1101–241,000	496–25,157	540–43,336
	st. dev. ^1^	4318	12,373	1696	2837
	cv % ^2^	38.3	123.7	29.8	69.7
	95 %	26,930	18,615	6120	5773
UFPs	average	10,183	9299	5271	3492
	min–max	2368–32,300	1031–251,438	467–23,382	493–41,990
	st. dev. ^1^	3810	12,137	1740	2550
	cv % ^2^	39.9	130.5	33.0	73.0
	95 %	16,472	25,348	6590	5160
no-UFPs	average	1722	707	424	580
	min–max	690–3664	70–12,522	31–8940	47–5170
	st. dev. ^1^	698	712	570	600
	cv % ^2^	40.5	100.7	173.0	128.2
	95 %	3172	1445	1500	1770

^1^ (st. dev. standard deviation) ^2^ cv % coefficient of variation (ratio between standard deviation and average × 100).
